# Application of coliphage as biocontrol agent in combination with gamma irradiation to eliminate multi-drug-resistant *E. coli* in minimally processed vegetables

**DOI:** 10.1007/s11356-023-31071-w

**Published:** 2023-11-23

**Authors:** Salwa A. Abou El-Nour, Ali A. hammad, Reham Fathy, Amal S. Eid

**Affiliations:** Radiation Microbiology Department, National Center for Radiation Research and Technology (NCRRT), Egyptian Atomic Energy Authority (EAEA), Cairo, Egypt

**Keywords:** Minimally processed vegetables, Anti-biofilm, Coliphage, *Escherichia coli*, Gamma irradiation, Combined treatment

## Abstract

Biofilm formation is a rising concern in the food industry*. Escherichia coli* (*E. coli*) is one of the most important food-borne pathogens that can survive in food and food-related environments and eventually produce biofilms. This study suggested that both coliphages used were successful in preventing the creation of new biofilms as well as removing existing ones. Confocal laser scanning microscopy verified these findings. According to the findings, neither coliphage survived at 37 °C, but both remained stable at 4 °C and − 20 °C for extended periods of time. The study revealed that both coliphages demonstrated a greater degree of gamma irradiation resistance when compared to *E. coli*. The study’s results indicate that the implementation of a dual method, which incorporates gamma irradiation (1.5 kGy) and coliphage treatment, on various kinds of vegetables that were infected with *E. coli*, resulted in a significant reduction in bacterial count (surpassing 99.99%) following a 24-h incubation period. Combining gamma irradiation and the coliphage approach was significantly effective at lowering polysaccharide concentrations and proteins in the biofilm matrix. The results revealed that the pairing of gamma irradiation and coliphages acted in conjunction to cause disruptions in the matrix of biofilm, thereby promoting cell removal compared with either of the individual treatments. Ca^+^ ions strengthen the weak virion interaction with the relevant bacterial host cell receptors during the adsorption process. In conclusion, use of coliphage in combination with gamma irradiation treatment can be applied to improve fresh produce’s microbial safety and enhance its storability in supermarkets.

**Graphical Abstract:**

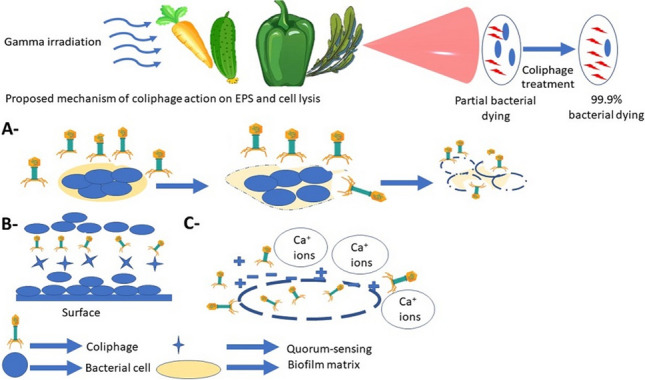

## Introduction

Fresh fruits and vegetables and their minimally processed products are commonly contaminated with many species of food-borne pathogenic bacteria, including multidrug-resistant and biofilm-forming ones. The presence of such pathogens in fresh produce is a serious health problem, causing many cases of food-borne illnesses and outbreaks since this fresh produce are usually consumed raw without additional heat treatment (Berger et al. 2010; Luna-Guevara et al. 2019). Fresh produce, namely, carrots, cucumbers, green peppers, and watercress, was selected due to their variety and correlation with outbreaks of food-borne diseases (Abadias et al. 2012; Litt et al. 2020; Snyder et al. 2016).

Food-borne illnesses have gained serious attention, with an estimated 420,000 deaths every year, 230,000 of which are related to diarrheal diseases (Havelaar et al. 2015). According to the World Health Organization (WHO) (https://www.who.int/health-topics/antimicrobial-resistance), antimicrobial resistance (AMR) is one of the top 10 worldwide public health concerns that humanity is currently facing in 2019. Approximately 73% of bacterial AMR deaths are attributed to six pathogens, with *Escherichia coli* (*E. coli*) at the top of the list, according to a large-scale systematic analysis of data from more than 200 countries conducted to assess the global burden of AMR (Murray et al. 2022).

*E. coli* is one of the most frequently encountered food-borne pathogenic bacteria, contaminating a variety of food products, including fruits, vegetables, beef chuck roast, ground beef, salmon, cheese, and fresh products (Sharma et al. 2009; Viazis et al. 2011; Kurtböke et al. 2016; Vikram et al. 2020; Puligundla and Lim 2022). Some strains of *E. coli*, such as *E. coli* O157:H7, are responsible for many cases of human illnesses and outbreaks (Puligundla and Lim 2022). Multi-antibiotic resistant *E. coli* has emerged in the last few decades due to the overuse and misuse of antibiotics (von Baum and Marre 2005). Several types of *E. coli* strains also have the capability to produce biofilms (Beloin et al. 2008).

Biofilms are a substantial problem in many food processing sectors, including dairy processing, seafood processing, meat processing, food brewing, and fresh produce (Srey et al. 2013). Biofilm is a structure of bacterial cells that attach to each other and to food surfaces embedded in a matrix of extracellular polymeric substances (EPS) containing polysaccharides, proteins, nucleic acids, and lipids. Such biofilm is a profoundly severe problem for food processing plants since it protects microbial cells against different traditional food treatments, including antimicrobial compounds (Costerton et al. 1999; Di Somma et al. 2020). In the food and healthcare industries, putrefying and pathogenic bacteria pose significant challenges due to their capacity to form biofilms, which protects them from routine cleaning techniques and prolongs their survival in the environment. By concentrating nutrients, blocking the entrance of biocides; isolating metals, and poisons; and preventing desiccation, extracellular polymers protect the inhabitants of biofilms. Thus, one aspect of the bacterial survival mechanism is the creation of biofilms. Nonetheless, the formation of biofilms by food-borne pathogens may significantly increase the chance of contracting food-borne diseases, posing serious health hazards to the population and having detrimental economic effects. Finally, it can be stated that biofilms are the main cause of food contamination, and their persistence on the surface and equipment that come into contact with food is the crucial factor that serves as an enduring source of contamination (Liu et al. 2023). Thus, conventional methods are insufficient for the elimination of biofilms due to their submersion in the matrix, which prevents antimicrobial substances from reaching biofilm-forming bacteria (Mittal et al. 2015). Various physical (heat), chemical sanitizers (peroxyacetic acid, chlorine dioxide, sodium hypochlorite, acidified sodium chlorite, organic acids, and aqueous ozone), and biological techniques have been used to regulate the existence of pathogenic bacteria in the food processing chain; nevertheless, their effectiveness has often been rather poor, and there are safety concerns (Gonzalez et al. 2004; Zhang et al. 2021; El-Shibiny 2016; Yoder et al. 2012). Furthermore, several pharmaceutical companies have drastically cut funding for research on antibiotics (Padmesh et al. 2023). Therefore, it is necessary to look for an effective and safer alternative treatment to eliminate biofilms and biofilm-forming bacteria. Combination therapy has shown promise in treating drug-resistant bacterial infections. Most of the time, it has had beneficial results. Examples of these combinations include the synergistic action of phages and antibiotics, as well as phages combined with probiotics (Osman et al. 2023). Among the most promising alternative ways are the combined treatment of bacteriophage and ionizing radiation.

The use of bacteriophages as an effective biocontrol agent for the removal or reduction of food-borne pathogenic and biofilm-forming bacteria in food products is a promising alternative due to their natural presence, being widely distributed, being environmentally friendly, being non-toxic to humans, being effective in bacterial cell lysis, and having high host specificity (Litt et al. 2020; Jagannathan et al. 2022). According to our knowledge, there are limited researchers interested in the elimination of multi-drug-resistant and biofilm-forming *E. coli* on the surfaces of minimally processed vegetables.

A growing number of phage preparations have received regulatory approvals, particularly those intended to improve food safety (Moye et al. 2018). The Food and Drug Administration (FDA) has approved the food safety products of several commercial phage companies, such as Intralytix, Micreos Food Safety, FINK TEC GmbH, Passport Food Safety Solutions, and Phagelux (Endersen and Coffey 2020). In line with the clearance of phage treatments, the FDA and the Food Safety and Inspection Service (FSIS) have approved several phage-containing products for commercial use that are available on the market to improve food products. These products include Listshield™, which was used in the USA against *Listeria monocytogenes* as a direct application on fish, processed fruits, processed vegetables, and dairy products (Moye et al. 2018; Perera et al. 2015). In addition, Vikram et al. (2020) conducted a thorough analysis of the Intralytix phage cocktail EcoShield PX™ in a meat system, focusing on *E. coli* that produces Shiga toxin. Nonetheless, the commercially available EcoShield PX™ (manufactured by Intralytix Inc.) showed superior results; on a range of food items, this preparation of phage cocktail was able to reduce *E. coli* O157:H7 levels by up to 97% in eight different food products, including ground beef, beef chuck roast, chicken breast, cooked chicken, romaine lettuce, cheese, salmon, and cantaloupe. The FDA in the USA and certain other nations (including Canada, Australia, and the European Union) have approved a number of phage-based products as generally recognized as safe (GRAS) for use in food safety applications (Cooper 2016; Moye et al. 2018).

Ionizing radiation (gamma irradiation, electron beams, or X-rays) has been proven to be highly effective in the elimination of food-borne pathogens contaminating food and their associated biofilms without compromising its chemical, physical, or nutritional value (WHO 1988; Farkas 1998; Munir and Federighi 2020). Recently, food irradiation technology has been applied in many countries and is approved by many international and national authorities, including World Health Organization (WHO), Food and Agriculture Organization (FAO), International Atomic Energy Agency (IAEA), CODEX Alimentarius Commission, Health Canada (HC), and the US-Food and Drug Administration (US-FDA) (WHO 1994; EFSA 2011).

Thus, the main goal of the present research is to evaluate the effectiveness of coliphages either alone or in combination with gamma irradiation on the multidrug-resistant and biofilm-forming *E. coli* artificially inoculated in minimally processed vegetables.

## Material and methods

### Bacterial strains and culture conditions

Two antibiotic-resistant *E. coli* isolates (*E. coli*-2 and *E. coli*-10) were previously isolated from different water sources in the Food Microbiology Laboratory, Microbiology Department, National Center for Radiation Research and Technology (NCRRT), Egyptian Atomic Energy Authority (EAEA), Cairo, Egypt (data not shown, work submitted for publication). Using the Charm Peel Plate EC Microbial Test (Kit Code: PP-EC-100k), *E. coli* was isolated and enumerated. This test is authorized as a performance-tested method by the Association of Analytical Communities (AOAC) research institution under License Number (061501/2021). In brief, each water sample was cultured for 18–24 h at 35 °C ± 1 on a Charm Peel Plate EC. *E. coli* was measured in colony-forming units (CFU/ml), which were round, blue, or black colonies. From every sample, one isolated *E. coli* colony was chosen. By streaking on eosin methylene blue agar (Oxoid, England) as a selective medium, these *E. coli* colonies were verified (Leininger et al. 2001). Each bacterial isolate was subcultured on LB agar plates for 24 h at 37 °C ± 1 to obtain pure colonies. To ensure the identification of *E. coli* isolates, the VITEK2 system, Version 08.01 (BioMerieux, Inc., Hazelwood, Mo. 63042), was used. *E. coli* isolates were used as coliphage hosts. Each isolate has been tested for its ability to form biofilm according to Freeman et al. (1989). They were cultured individually in tryptic soy broth (TSB, Oxoid) supplemented with CaCl_2_ for 24 h under aerobic conditions at 37 °C ± 1 in a shaking incubator (120–150 rpm) to reach an optical density OD_600_ of 0.5 (corresponding to the log-phase of growth of 10^8^ CFU/ml was used for each assay carried out in this study). Bacterial cultures were kept in TSB at 4 °C ± 1. These isolates were preserved in glycerol at − 80 °C for further usage.

Antimicrobial sensitivity tests were performed to determine whether two *E. coli* isolates were resistant to different antibiotic classes by using the disc diffusion technique as described by Humphries et al. (2021). In this experiment, eight antibiotic classes were used: aminoglycosides (streptomycin (10 µg), neomycin (30 mcg)); cephalosporins (ceftriaxone (30 mcg), ceftazidim (30 µg), cefotaxime (30 µg), cephalexin (30 mcg)); phenicols (chloramphenicol (30 mcg)); β-lactams (AMC-30); sulfonamides (trimemethoprim/sulfamethoxazole); aminopenicillins (ampicillin (10 mcg)); carbapenems (imipenem (10 mcg)), and fluoroquinolone (Nalidixic acid (30 mcg)). *E. coli*-2 showed resistance to five of the 12 tested antibiotics (ceftriaxone (30 mcg), ceftazidim (30 µg), AMC-30, cefotaxime (30 µg), and cephalexin (30 mcg). While *E. coli*-10 resisted seven out of the 12 tested antibiotics (streptomycin (10 µg), AMC-30, cefotaxime (30 µg), trimemethoprim/sulfamethoxazole, ampicillin (10 mcg), nalidixic acid (30 mcg), and cephalexin (30 mcg).

### Coliphages preparation

Two tested bacteriophages (Somatic coliphage, S3 belongs to the *Myoviridae* family, and F-specific, F3 coliphage belongs to the *Levivivirdae* family) were earlier isolated from ground water (Assiut, Egypt) and aerobic-activated sludge (El-Gabal El-Asfar Stage-2, Cairo government, Egypt domestic wastewater treatment plant), respectively, distinguished, and tested to investigate their lytic activity against *E. coli*-2 and *E. coli*-10, respectively (data not shown, work submitted for publication). Two tested coliphages were chosen for application to minimally processed vegetables based on their lytic activity. Lytic activity of isolated coliphages was checked against the 12 *E. coli* isolates to evaluate the host activity of each coliphage by using a double-layer plaque (DLA) assay. The coliphages were enumerated with their host *E. coli* by counting the plaques that represent patches of dead bacteria and one virus apiece, according to Sambrook and Michael (2012). As described by Oliveira et al. (2016), phages were propagated. Plaque assays, according to Adams (1959), were used to measure phage titers as plaque-forming units (PFU/ml) before and during an experiment. High-titer lysates of the phage (10^10^ PFU/ml) were prepared as described by Swanstrom and Adams (1951) using TSB media supplemented with 1 M CaCl_2_ (10 ml/l).

### Coliphage isolation and purification

The previously mentioned water samples were used to isolate somatic and male-specific coliphages (F-specific coliphages) using Charm Sciences Fast Phage EPA test kits (FP-SOM-25K and FP-FPLUS-25K), which are equivalent to USEPA Method 1601. After removing any leftover bacterial cells with sterile 0.22-µm diameter pore-size membrane syringe filters (CHROMAFIL® Xtra PES, 20–25 mm, item number: 729012 Macherey–Nagel GmbH&Co.KG, Germany), the double-layer agar (DLA) method was used to determine whether bacteriophage was present in the final filtered liquid. In phosphate buffer solution (PBS), serial dilutions (10^1^–10^10^) of each coliphage in its final filtered liquid were prepared (Yang et al. 2010). One milliliter of host strain (*E. coli*) was added to each 1 ml of these dilutions, and they were incubated for 20 min at 37 °C at 120 rpm. Each dilution was then mixed with 3 ml of 0.7% top layer molten agar and transferred onto Petri plates that contained solid tryptone soy medium. After overnight incubation, the plates were examined for the existence of phage plaques.

Purification of the isolated coliphages was accomplished according to the method described by Maszewska et al. (2016). A single plaque was picked up and transferred to the TSB containing the specific host bacteria for 3 h. The phage suspension was centrifuged, and the supernatant was filtered to remove any remaining bacterial cells. To confirm that the isolated phage was the progeny of a single virion, this procedure was repeated three times. The purified coliphages were stored at 4 °C for a short time and at − 20 °C for a longer period (6 months). The phages’ high-titer lysates (10^10^ PFU/ml) were prepared as directed by Swanstrom and Adams (1951). The phage susceptibility and purity were evaluated using a spot assay (Kutter 2009). Clearance of bacterial growth at the spot position indicates the presence of a single type of coliphage specific to a single type of inoculated bacteria.

### Phenotypic analysis of EPS production

By cultivating the tested bacterial strains on Congo red agar (CRA), a modified method of Freeman et al. (1989) was used to assess the *E. coli* isolate capacity to produce exopolysaccharide (EPS). Exopolysaccharide production can be determined by evaluating changes in colony color. According to Arciola et al. (2002), the current study classified colony colors precisely using a six-color reference scale. The three results that were rated as negative on the scale were bordeaux, red, and very red. The three outcomes that were deemed positive were very black, black, and almost black. Three triplicates of the experiment were performed.

### Anti-biofilm activity of coliphage

The potential of bacteriophages for inhibiting the tested biofilm forming *E. coli* was estimated, according to Stepanovic et al. (2000). Twenty microliters of each tested *E. coli* strain (10^8^ CFU/ml) was individually inoculated in LB broth and distributed to wells in 96-well flat-bottom microtiter plates (Thermo-Fisher Scientific) as a positive control for the biofilm formation inhibition experiment. The wells were filled with 20 µl of *E. coli*-2 mixed with 20 µl of S3 coliphage (10^10^ PFU/ml) (MOI = 1) and 20 µl of *E. coli*-10 mixed with 40 µl of F3 coliphage (10^10^ PFU/ml) (MOI = 2). The relative abundances of *E. coli* found in an earlier study (data not shown, work submitted for publication) were used to determine the bacteriophage MOI. Microplates were aerobically incubated for 24 h at 37 °C. To remove any planktonic cells, the contents of the plate were thoroughly rinsed three times with sterile distilled water. The wells were fixed with methanol and air-dried after that. The microplates were stained with crystal violet (1%) for 5 min. Using a TECAN Sunrise Microplate Reader tool and a wavelength of 660 nm, *E. coli* biofilm values were calculated using the optical density (OD) of each well. Based on their OD values, the strains were categorized into three groups: low, mild, and strong biofilm former. Based on the cut-off OD (ODc), which is three standard deviations above the mean OD of the negative control (Stepanović et al. 2004), isolates can be categorized into three groups: weak, moderate, or strong biofilm formers. The categories for isolates were as follows: ODc < OD ≤ (2 × ODc) = weak biofilm producer, 2ODc < OD ≤ (4 × ODc) = moderate biofilm producer, and (4 × ODc) < OD = strong biofilm producer. Subsequently, OD ≤ ODc indicates no biofilm producer. A previously established process was used to create the turbidity analysis settings (Vengarai Jagannathan et al. 2020). For a visual representation of the phage anti-biofilm activity against *E. coli*, the OD_660_ values were plotted against time. The broth media was used as negative control.

### Biofilm degradation by coliphages

According to the methods described by Gharieb et al. (2020), the biofilm degradation experiment was conducted. Two hundred microliters of both overnight tested *E. coli* suspensions (10^8^ CFU/ml) were initially incubated under the previously mentioned conditions for 48 h to allow biofilm formation. Then, 200 µl and 400 µl of bacteriophage culture (10^10^ PFU/ml) S3 coliphage and F3 coliphage were added, respectively. The mixture was incubated for 24 h at 37 °C. Crystal violet was used to measure the removal of biofilm after the duration of the exposure, as previously mentioned. As a control, a procedure without phages was used.

### Evaluating of the persistence of biofilms after phage treatment

A confocal laser scanning microscope (CLSM) was used to evaluate the persistence of biofilms after phage treatment. To differentiate between live and dead bacteria in biofilms after phage treatments, the Film Tracers Live/Dead® Biofilm Viability Kit (Molecular Probes®, USA) was used in accordance with the manufacturer’s instructions. The LIVE/DEAD BacLight Bacterial Viability Kit is supplied with both the SYTO-9 green, fluorescent nucleic acid stain, and the propidium iodide red fluorescent nucleic acid stain, which are employed to distinguish between viable and non-viable bacteria, respectively. A confocal laser scanning microscope (Leica DMi8 CEL, Germany) located at National Research Center, El Dokki, Egypt, with a 40 × objective lens and a laser operating at 488 nm for excitation (emission, 500 to 550 nm) was used to study the development of static biofilms. In every experiment, the microscopic examination of at least 10 randomly chosen fields from four different cultures was used.

### Lytic efficacy of coliphages in the 1 month

The purified S3 and F3 coliphages (10^10^ PFU/ml) were incubated at freezing temperature (− 20 °C), refrigeration temperature (4 °C), and ambient temperature (37 °C) for a whole month, followed by an assay of phage activity according to Adams (1959) to investigate phage viability.

### Irradiation process

The gamma irradiation source used is an Indian Co-60 Gamma Chamber 4000A, located at the NCRRT, EAEA, Nasr City, Cairo, Egypt. This radiation source’s dose rate was 0.717 kGy/h. Processes involving irradiation were conducted at room temperature. By altering the exposure time, different gamma doses were obtained. In three separate experiments, all samples underwent three radiation exposures. Alanine dosimeters (Traceable to National Physical Laboratory, UK) were used for dose calibration of the source and for measuring the average absorbed dose. Detailed dose mapping was achieved by the Department of Radiation Protection and dosimeter according to the Egyptian Standards.

### Calculation of D-_10_ values

The linear regression model of the log of the surviving fractions was used to calculate D-_10_ values in kGy, where it is defined as the amount of radiation dose needed to kill 90% of the microorganisms. The D-_10_ value for each microbe was determined by utilizing the slope of the dose–response curve, which was generated by plotting log survival counts against the applied irradiation doses. The slope was calculated through a linear regression analysis utilizing Microsoft Office Professional’s Excel software, as per the following formula:

$$\begin{array}{cc}{D}_{10}Value=-\frac{1}{b}& b=\frac{\sum xy-n xy}{\sum {x}^{2}-n {x}^{2}}\end{array}$$

where *x* = dose level (kGy), *y* = logarithmic survival rate following *x* dose of radiation, and *n* = number of calculated points

### D-_10_ values of both *E. coli* and coliphages

In screw-cap test tubes, 10 ml of the *E. coli* log-phase culture cell suspension (10^8^ CFU/ml) from each tested strain was added. The test tubes were subsequently subjected to gamma radiation at various doses (0.0, 0.5, 1.0, 1.5, 2.0, and 3.0 kGy) with exposure times (0, 41.8, 83.6, 125.5, 167.3, and 251.0 min), respectively. Three tubes (in triplicate) were used for each dose. Ten milliliters of each pure coliphage suspension (10^10^ PFU/ml) was subjected to five various doses of gamma irradiation (0.0, 1.0, 2.0, 3.0, 4.0, and 5.0 kGy) with exposure times (0, 41.8, 83.6, 125.5, 167.3, 251.0, 334.7, and 418.4 min), respectively. For each dose, three tubes (in triplicate) were used.

### Enumeration of *E. coli* and coliphages survivors

The survivors of *E. coli* following each radiation dose were enumerated using standard pour plate technique using tryptic soy agar medium (TSA, Oxiod) and incubated at 37 °C for 24–48 h. The quantity of colony-forming units (CFU)/ml was subsequently determined. Following irradiation, the number of coliphage survivors was determined according to Adams (1959), and the double-layer agar method was used to count the survivors (PFU/ml).

### Preparation of minimally processed vegetables

Fresh produce, namely, carrots, cucumbers, green peppers, and watercress, were selected due to their variety and correlation with outbreaks of food-borne diseases. They were bought from the local fruits and vegetables market in Cairo on the same day of the experiments, transferred in ice box, and preserved at 4 °C until use. The fresh produce was carefully washed under running water, allowed to air dry for 30 min, and exposed to a UV lamp for 30 min to reduce and eradicate any natural bacteria. The produce was further prepared as follows: fresh cucumbers and carrots (5 kg of each) were chopped down into circular slices with a fixed surface area of 5 cm^2^, green papers (5 kg) were manually divided into quarters, and leaves and stalks of watercress were trimmed. All samples were packed in 100-g foam plates (10 × 10 cm) and wrapped with high transparency, self-clinging film after treatment.

### Application experiments

To test the effects of gamma irradiation, coliphages, and irradiation combined with coliphages, each of the four types of prepared vegetables was split into eight experimental groups:

The 1st groups of minimally processed vegetables were immersed individually in 100 ml of *E. coli*-2 cell suspension (10^8^ CFU/ml) for 10 min. In the same manner, the 2nd group of samples was artificially inoculated with 100 ml of *E. coli*-10 (10^8^ CFU/ml) for 10 min. Both the 1st and 2nd groups were allowed to air-dry for 1 h in a sterilized cabinet, transferred to sterilized foam plates, and covered with wrapped high transparency, self-clinging film and used as controls. For post-contamination treatment studies, samples from the 3rd and 4th groups were contaminated by soaking them for 10 min in 100 ml of *E. coli*-2 cell suspension and 100 ml of *E. coli*-10, respectively. All the slices were then taken out and dried. After being packaged, they were treated with 2.0 kGy of gamma radiation with exposure time (167.3 min).

The 5th group samples were immersed in 100 ml of *E. coli*-2 cell suspension for 10 min, followed by immersed treatment with 100 ml of S3 coliphage (10^10^ PFU/ml) (MOI = 1) for 30 min. The 6th group samples were contaminated with 100 ml of *E. coli*-10 for 10 min, followed by treatment with 200 ml of F3 coliphage (10^10^ PFU/ml) (MOI = 2) for 30 min.

The 7th group samples were dipped in 100 ml of *E. coli*-2 for 10 min followed by treatment with somatic coliphage (S3) (10^10^ PFU/ml) (MOI = 1) for 30 min, packaged and irradiated at 1.5 kGy. In the same manner, the 8th group samples were artificially contaminated with 100 ml of *E. coli*-10 for 10 min, irradiated at 1.5 kGy, after treated with 200 ml of F3 coliphage (10^10^ PFU/ml) (MOI = 2) for 30 min, and packaged. After treatments, all group samples were sealed with parafilm in plastic Petri dishes and stored at 4 °C for 9 days. Treated samples were periodically taken after (1, 3, 6, and 9 days) to enumerate *E. coli* survivors (CFU/g), as mentioned before. Three replicates were used every time.

### Biofilm analysis

#### EPS and surface-bound proteins in the biofilm matrix

The effects of bacteriophage, gamma irradiation (1, 1.5, and 2.0 kGy), and combined treatment (coliphage + 1.5 kGy) on the EPS of the host strain were estimated. The treated bacterial cells had been cultured in LB media for 24 h at 37 °C before being centrifuged for 15 min at 4 °C at 4000 rpm. According to Nehad and El-Shamy (2010), the culture filtrate was combined with four volumes of pure ethanol and kept at 4 °C for 24 h to precipitate the crude EPS. The phenol–sulfuric acid method, according to Chaplin and Kennedy (1986), was used to measure the EPS yield after the lyophilization of precipitated EPS. A glucose standard curve was used to detect an unknown concentration, with absorbance measurements taken at a wavelength of 490 nm.

Surface-bound proteins have been extracted using a modified version of Castellanos et al.’s (1997) procedure. According to the Lowry et al. (1951) method and bovine serum albumin (BSA) as a reference, the protein content of the supernatant was assessed.

#### Zeta potential and dynamic light scattering (DLS)

For both tested *E. coli* strains, *E. coli* + coliphage after (0, 4 h), *E. coli* + gamma irradiation (1.5 kGy), and combined treatment (*E. coli* + coliphage + gamma irradiation (1.5 kGy), the bacterial cell surface charge and cell size distribution profile were detected using a Zeta potential/particle sizer (NICOMP380 ZLS, PSS. NICOM Particle sizing systems, Santa Barbara, California, USA) at NCRRT. The red He–Ne laser diode’s incident light had an applied wavelength of 632.8 nm. At 23 °C, measurements were carried out. Three separate zeta potential measurements were averaged to produce the data.

### Statistical analysis

Each experiment was established at least three times, and the results were plotted using the mean ± SE. A one-way ANOVA was used to statistically analyze the data for this study (Minitab 19.1.1, Minitab LLC, UK). The highest value is symbolized by (a) letter, while small letters denote relevance between groups. Linear regression comparison tests were used to compare statistical data for the different microorganisms and matrices; the data were deemed statistically significant at a *P*-value < 0.05.

## Results

### Phenotypic analysis of EPS production

**Fig. 1:**
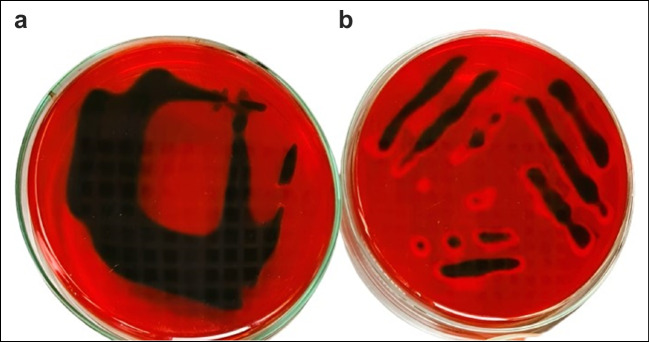
Screening of biofilm producers by Congo red agar medium: **a** *E. coli*-2 (very black colonies) and **b** *E. coli*-10 (black colonies)

Both tested *E. coli* isolates produced black, opaque colonies on Congo red agar (CRA) within the first 24 h of incubation, demonstrating their capacity to synthesize EPS. Figure 1 illustrates that the red media turned black, which is a positive result. The obtained results indicated that the tested *E. coli*-2 isolate is a strong biofilm former bacterium, while *E. coli*-10 is a moderate biofilm former bacterium, according to the colorimetric scale suggested by Arciola et al. (2002).

### Anti-biofilm activity and established biofilm degradation by coliphages

**Fig. 2:**
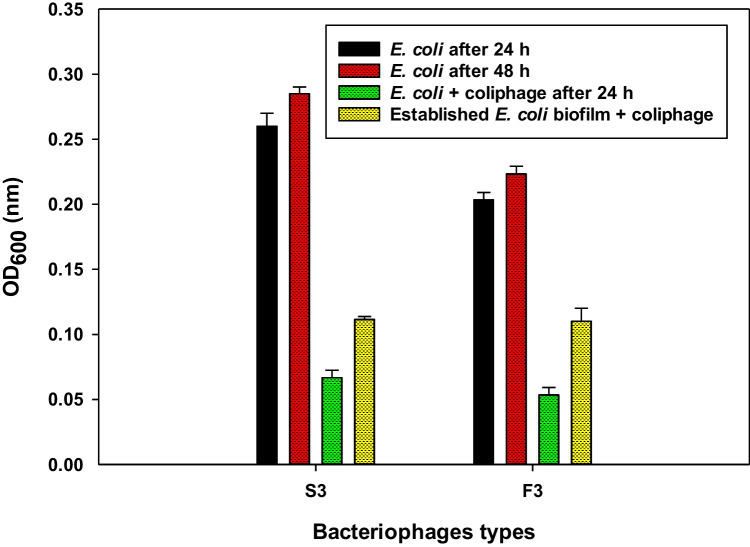
Biofilm growth inhibition activities of tested *E. coli* isolates and reduction of established *E. coli* biofilm by lytic coliphages (S3 and F3)

The optical density of both untreated *E. coli*-2 and *E. coli*-10 application in biofilm grown in 96-well microtiter plates at 37 °C ± 1 for 24 and 48 h is shown in Fig. 2. In comparison with control, the results indicated that when each coliphage was primary inoculated with its specific *E. coli* at zero time, they are significantly reduced the growth activity of bacterial cells from 0.26 ± 0.010^b^ and 0.20 ± 0.005^d^ to 0.06 ± 0.005^f^ and 0.05 ± 0.005^f^ for *E.coli*-2 and *E. coli*-10, respectively, and these results were obviously observed after only 24 h. Therefore, it could be concluded that they have the ability in preventing the formation of bacterial biofilms. S3 coliphage reduced the growth activity of *E. coli*-2 by 76.9% from the initial growth activity of control at 24 h. Meanwhile, F3 reduced the growth activity of *E. coli*-10 by 75%.

On the other hand, it could be observed that the effect of each coliphage on already established (grown) biofilm is less than its effect on primary inoculation. It could be observed that S3 coliphage reduced the developed (established) *E. coli*-2 biofilm biomass by 60.7%, while coliphage F3 resulted in 50% reduction in the formed biofilm. These results indicated that both coliphages not only efficiently removed a bacterial biofilm that had already formed but also stopped it from growing.

### Evaluating the persistence of biofilms following phage treatment using confocal laser scanning microscope

**Fig. 3:**
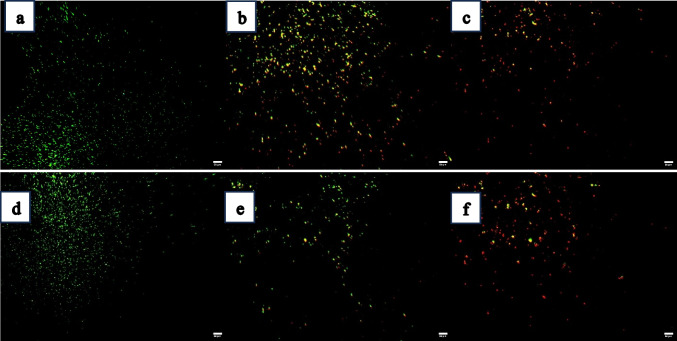
Live/dead confocal laser scanning microscopic (CLSM) images of biofilms treated with phage. Green color indicated live cells and red color for dead cells. **a** Biofilm of untreated *E. coli*-2 (control), **b** biofilm of *E. coli*-2 exposed to phage for 0 min, **c** biofilm of *E. coli*-2 exposed to phage for 4 h, **d** biofilm of untreated *E. coli*-10 (control), **e** biofilm of *E. coli*-10 after a 0 min phage exposure and **f** biofilm of *E. coli*-10 after 4 h of phage treatment. The scale bar is 50 μm

Under confocal laser scanning microscope, two different bacterial cells can be distinguished: green for viable cells and red for harmed or non-viable cells. Figures 3a and d represent the dense green coloration of an untreated biofilm for *E. coli*-2 and *E. coli*-10, respectively. Immediately, after treatment with coliphages, the treated *E. coli*-2 and *E. coli*-10, respectively, showed a clear matrix of green cells with few red cells (dead cells) as shown in Fig. 3b and e. Very few cells were still viable after 4 h of coliphage treatment due to cell lysis as represented in Fig. 3c and f.

### Lytic efficacy of coliphages in the 1 month

**Fig. 4:**
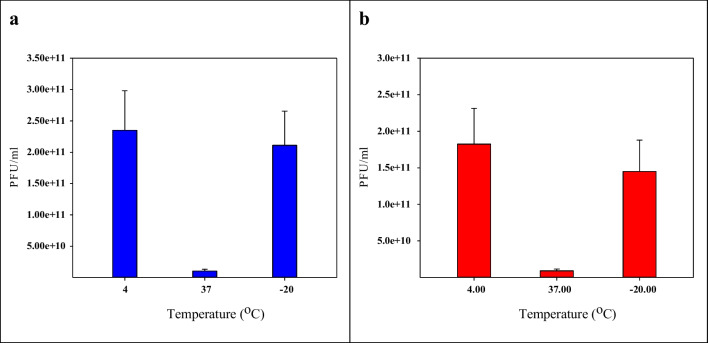
The average PFU/ml of coliphages after incubation for a month depending on the storage temperature (− 20 °C, 4 °C, and 37 °C): **a** S3 coliphage, **b** F3 coliphage

The purified coliphages S3 and F3 (10^10^ PFU/ml) were tested for viral titers after 1 month of incubation at various temperatures (4, 37, − 20°C). At 4 and − 20 °C both coliphages, lytic activity almost remained stable. Both S3 and F3 coliphages showed maximum activity against *E. coli* at 4 °C, while at 37 °C, the lytic activity of both was the least as observed in Fig. 4.

### Radiation response (D_10_-values) of the tested *E. coli* and coliphages

**Fig. 5:**
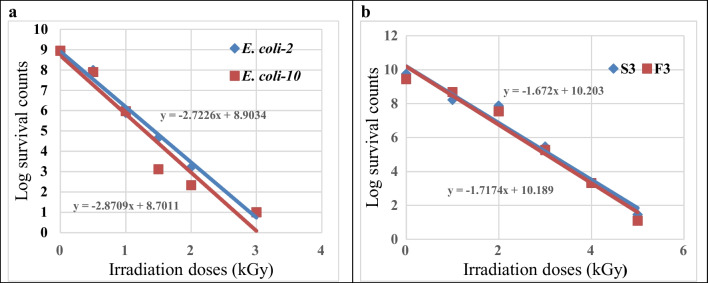
Determination of gamma irradiation dose response curve of **a** both *E. coli*-2 and *E. coli*-10 and **b** both S3 and F3 coliphages

D_10_-values of *E. coli-*2 and *E. coli*-10 ranged between 0.34 and 0.36 kGy (Fig. 5a). This indicates the gamma irradiation dose required to reduce the number of both *E. coli* by 5-log_10_ is 1.75 kGy. Radiation dose response curves of both somatic coliphage and F-specific coliphage were also exponential (Fig. 5b) and their D_10_-values were 0.598 and 0.582 kGy, respectively. This indicates that their response to gamma radiation was almost similar, and they were more radio-tolerant in comparison with their *E. coli* hosts.

### Experimental applications

**Fig. 6:**
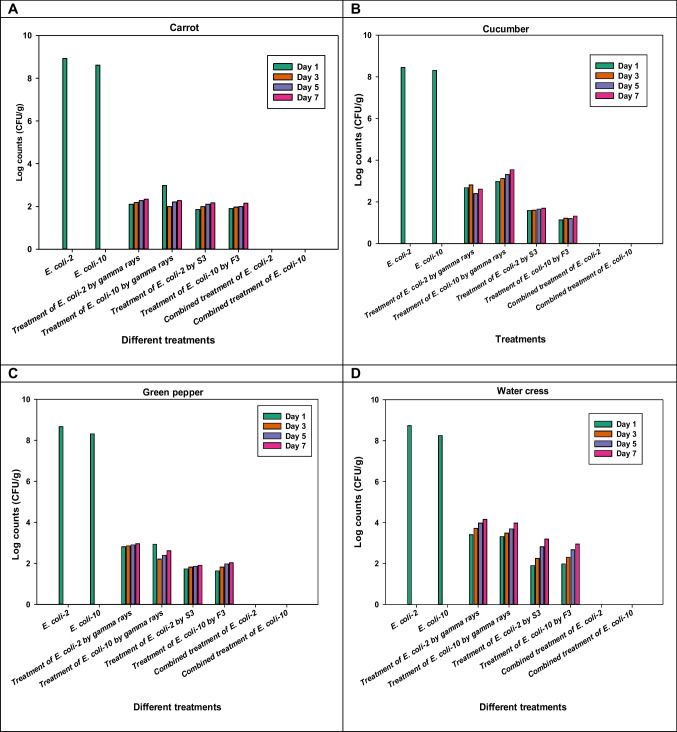
Effect of gamma irradiation, coliphages, and their combination treatments on the artificial contamination by both tested *E. coli* on **a** carrot; **b** cucumber; **c** green pepper; and **d** water cress

When carrot slices were treated with gamma irradiation (2.0 kGy), the log counts of *E. coli*-2 and *E. coli*-10 dropped by 6.76 and 6.62 on the third day of storage at 4 °C, respectively. This can be seen in Fig. 6 and Table 1. The contaminated carrot slices were also treated with both S3 and F3 coliphages, which led to a higher log count reduction of *E. coli*-2 and *E. coli*-10 than gamma irradiation (7.09 and 6.72 log counts reduction, respectively).

**Table 1:** Log counts (CFU/g) of treated contaminated carrots, cucumber, green pepper, and watercress at various storage times (days) at 4 ℃ using gamma irradiation, coliphages, and combined treatment

		Carrot (Log counts)		Cucumber (Log counts)		Green pepper (Log counts)		Watercress (Log counts)	
		*E. coli*-2	*E. coli*-10	*E. coli*-2	*E. coli*-10	*E. coli*-2	*E. coli*-10	*E. coli*-2	*E. coli*-10
Control (4 h) 4 °C		8.94	8.62	8.45	8.31	8.67	8.32	8.73	8.25
Treatment with gamma irradiation (days)	1	2.9	2.98	2.68	2.98	2.81	2.93	3.41	3.32
	3	2.18	2	2.81	3.12	2.85	2.21	3.72	3.49
	6	2.27	2.21	2.4	3.31	2.9	2.4	3.98	3.69
	9	2.34	2.27	2.61	3.38	2.96	2.62	4.15	3.98
Treatment with S3/F3 coliphages (days)	1	1.85	1.9	1.59	1.13	1.73	1.63	1.9	1.98
	3	1.99	1.97	1.6	1.21	1.82	1.82	2.25	2.31
	6	2.1	2.03	1.65	1.2	1.86	1.97	2.82	2.67
	9	2.17	2.14	1.69	1.32	1.91	2.02	3.19	2.98
Combined treatment (Gamma rays (1.5kGy) + either S3 or F3 coliphages)		< 10 CFU/g	< 10 CFU/g	< 10 CFU/g	< 10 CFU/g	< 10 CFU/g	< 10 CFU/g	< 10 CFU/g	< 10 CFU/g

Additionally, from both Fig. 6 and Table 1, it could be revealed that the application of gamma irradiation (2.0 kGy) to contaminated cucumber led to a dramatic decrease in the log counts of *E. coli*-2 and *E. coli*-10 by 6.05 and 5.33 on the sixth and first days of storage at 4 °C, respectively. Conversely, after 24 h of storage at 4 °C, the application of S3 and F3 coliphages to treat the contaminated cucumber led to a higher reduction in the log counts of *E. coli*-2 and *E. coli*-10 than gamma irradiation, which produced log count reductions of 6.86 and 7.18, respectively.

It is possible to infer from Fig. 6 and Table 1 that the contaminated green pepper treated with gamma irradiation (2.0 kGy) saw a significant reduction in *E. coli-*2 *and E. coli-*10 log counts on the first and third days of storage at 4 °C by 5.86 and 6.11, respectively. In contrast, the use of S3 and F3 coliphages to treat the contaminated green pepper after 24 h of storage at 4 °C resulted in a greater decrease in the log counts of *E. coli*-2 and *E. coli*-10 than gamma irradiation, which generated log count reductions of 6.94 and 6.69, respectively.

Furthermore, Fig. 6 and Table 1 show that, after 1 day of storage at 4 °C, the quantity of *E. coli*-2 and *E. coli*-10 in contaminated watercress decreased by 5.32 and 4.93 log reductions, respectively, after exposure to gamma irradiation at 2.0 kGy. In addition, after 1 day of incubation at 4 °C, the S3 and F3 coliphages reduced the *E. coli*-2 and *E. coli*-10 log counts by 6.83 and 6.27 logs, respectively.

Extending the storage period at 4 °C does not significantly affect the reduction of log counts, according to the data gathered from all minimally processed foods that were examined. The most significant results were obtained from the combined treatment of gamma irradiation (1.5 kGy) and coliphage (either S3 or F3), which reduced both tested strains of *E. coli-*2 and *E. coli*-10 to undetectable levels (< 10 CFU/g) throughout the storage period.

### Biofilm analysis

#### EPS and proteins in the biofilm matrix

**Fig. 7:**
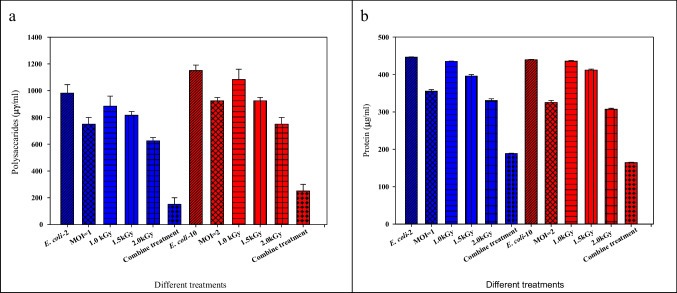
Effect of the different treatments on **a** polysaccharides and **b** proteins

Polysaccharides and proteins in the EPS fraction of the biofilm were quantified under different conditions. All treatments (coliphages, gamma irradiation (1, 1.5, and 2.0 kGy), and combined treatment (coliphage + 1.5 kGy) caused a reduction in both biofilm-associated polysaccharides and proteins compared to the control. Gamma irradiation treatment (2.0 kGy) resulted in a reduction of polysaccharide and protein concentrations by 36.2, 25.8% for *E. coil*-2 and 34.7, 29.8% for *E. coli*-10, respectively. The combined treatment of gamma irradiation (1.5 kGy) and coliphages reduced at least 84.6% of polysaccharides and 57.7% of protein concentrations for *E. coil*-2 and 78.2 and 62.2% for *E. coli*-10, respectively, in comparison with the control (Fig. 7). Gamma irradiation (2.0 kGy) combined with coliphages treatment achieved a statistically higher polysaccharide and protein reduction compared with coliphage treatment alone.

### The alteration of surface charges of coliphages

The addition of Ca^+^ ions, which act as bridges, enables many negatively charged phage virions to interact more forcefully with bacterial cells. Alteration of surface charges of a coliphage promotes its sensitivity. Hence, the surface charges of *E. coli* were measured using zeta potential measurements to investigate the electrostatic charges on the surfaces of untreated and treated bacterial cells. The exposure of both tested *E. coli* isolates to gamma irradiation significantly changed the cell surface charge, resulting in increased cell negativity. Cell negativity decreased because of the combined treatments. Interesting findings are the effects of bacteriophage on *E. coli*, which resulted in a decrease in cell negativity from − 12.15 to − 0.01 mV and from − 15.8 to − 1.08 mV for S3 and F3 coliphages, respectively, as shown in Table 2. In addition, from this table, it could be observed that cell radius decreased when bacterial cells were treated with bacteriophages as well as combined treatments. On the other hand, gamma irradiation resulted in increased bacterial cell size.

**Table 2:** Dynamic light scattering (DLS) and zeta potential of untreated and treated *E. coli*

Parameters	DLS (nm)	Zeta potential (mV)
*E. coli*-2	1536.0	− 12.15
1:1 (0 time)	1453.0	− 7.8
1:1 (4 h)	278.9	− 0.01
1.5 kGy	546.5	− 20.1
Combine treatment	277.1	− 8.02
*E.coli*-10	1761.3	− 15.8
1:2 (0 time)	1529.3	− 14.17
1:2 (4 h)	371.4	− 1.08
1.5 kGy	566.2	− 23.44
Combine treatment	271.7	− 11.13

## Discussion

One of the major challenges facing minimally processed vegetables is their contamination with food-borne pathogenic bacteria, including multi-antibiotic and biofilm forming *E. coli*. A bacterial biofilm is a group of microbes that can adhere to surfaces and create an extracellular matrix and exopolysaccharide that is helpful for the survival and pathogenicity of bacteria. According to the findings of the present study, both the tested multi-antibiotic resistance *E. coli* isolates were found to produce biofilms. Gonçalves et al. (2017) clarified the relationship between biofilm production and antibiotic resistance, which makes it difficult for drugs to pass through the outer membrane of bacteria, where they secrete a polymeric matrix made up of proteins, DNA, and polysaccharides that serve as a physical barrier to drug entry. It is therefore necessary to use a novel alternative treatment to reduce the use of antibiotics such as bacteriophages and irradiation.

Regarding the coliphages ability to prevent *E. coli* isolates from forming biofilms, the tested coliphages revealed highly preventive activity within 24 h when compared with the control (without phage), where both coliphages reduced the growth rate of *E. coli*-2 and *E coli*-10 by 76.9% and 75%, respectively. This prevention may be due to the production of phage enzymes, which reduce the capacity of bacteria to form biofilms; this theory is in line with that of Hanlon (2007) who stated that numerous nucleotides in the phage DNA are chemically altered to provide defense against harm by nuclease enzymes and cellular restriction. The beneficial impact of bacteriophage interaction with bacterial biofilms in the prevention of biofilm formation was also established by Sulakvelidze et al. (2001) who clarified that therapeutic phages are believed to have lytic activity against target bacteria. The lytic process is complicated and involves several structural and regulatory genes, as shown by T4 phage replication.

Meanwhile, after 48 h, the results illustrated that both coliphages were effective in removing established (grown, developed) bacterial biofilms by 60.7% and 50% for *E. coli*-2 and *E. coli*-10, respectively, possibly through the enzymatic activities that allow these coliphages to effectively penetrate biofilms and degrade them. This suggestion agrees with Knirel et al. (2020), who claimed that during the phage-bacterium interaction, phages produce polysaccharide depolymerase that decomposes the extracellular polymeric material in the biofilm, thereby allowing phages to penetrate encapsulated bacterial cells and cause lysis. Contrary to antibiotics, which only affect bacteria on the surface of the biofilm, bacteriophages can enter the biofilm’s inner layer. Additionally, persisted cells can be infected by bacteriophages and destroyed if they are reactivated. By creating an enzyme or by encouraging the bacterial host to produce an enzyme, they can also dissolve the biofilm matrix (Lewis 2007).

Consequently, the results obtained could indicate that coliphages can indirectly lyse biofilms by killing bacteria either before or after they colonize on the surface. Phages are more efficient at removing biofilm than antibiotics, according to Morris et al. (2019), who clarified that within 8 h of the bacterial injection, staphage therapy reduced *Staphylococcus aureus* growth by 98%. As well as confocal laser scanning microscope image also confirmed the effect of bacteriophage on bacterial cell.

According to standard storage conditions of minimally processed vegetables, coliphage stability was estimated during refrigeration, freezing, and ambient storage conditions to reduce *E. coli* contamination in foods. According to the data, both coliphages remained stable at 4 °C and − 20 °C for 1 month, while none survived at 37 °C. These findings are consistent with Khawaja et al. (2016) who stated that the TSE bacteriophages remained stable at low temperature for a month, while none survived at 37 °C. These results may have significant practical implications during food storage at low temperatures. For example, fresh vegetables and other foods may be sprayed with phages during different postharvest and processing stages, e.g., before packaging and transport or during storage in the refrigerator.

The consumption of minimally processed vegetables has been increasing interest in the last decades because of change in lifestyle and convenience of these products. However, these products can be contaminated with a variety of food-borne pathogenic bacteria during pre-, post-harvesting as well as during processing (peeling, slicing, cutting, or shredding). As these fresh minimally processed vegetables are eaten raw without further heat treatment, their contamination by food-borne-pathogenic bacteria is of particular importance from the viewpoint of public health (Beuchat 2002). The traditional methods commonly used for disinfection of these products such as washing, chlorination, or UV-irradiation are not sufficient and leave harmful residues. Thus, there is an increasing interest in recent years to use alternative disinfection effective methods. Gamma irradiation is proven to be very effective technology in preserving food and elimination of food-borne-pathogenic bacteria present in food and food products including minimally processed vegetables (WHO 1988; Farkas 1998; Agbaka and Ibrahim 2020). Recently, the effectiveness of bacteriophages as a biocontrol agent for food preservation and reducing food-borne-pathogenic bacteria has been reported (Litt et al. 2020; Gildea et al. 2022). Therefore, the main objective of the present study is to combine gamma irradiation and coliphages for elimination of multi-drug-resistant and biofilm-forming *E. coli* artificially inoculated into slices carrots, slices cucumber, quarter green pepper, and trimmed watercress. *E. coli*-2 and *E. coli-*10 were previously isolated from different wastewater. Somatic coliphage targeting *E. coli*-2 and F-specific coliphage targeting *E. coli*-10 were used.

The response (resistance or sensitivity) of both *E. coli* and coliphage to gamma irradiation was determined in the form of D_10_-value which is defined as irradiation dose required to inactivate 90% of a microbe population or reduce its population by one log_10_ cycle. The result obtained in our study indicates that the D_10_-values of *E. coli*-2 and *E. coli*-10 were 0.36 and 0.34 kGy, respectively. This range of D_10_-values was within the range of D_10_-values previously reported by many investigators, who found that the D_10_-values of *E. coli* ranged between 0.20 and 0.42 kGy (Farkas 1998; Abu El-Nour Salwa 2007). The small differences of the D_10_-values within the same bacterial species could be due to the different strains.

The susceptibilities of somatic-coliphage and F-specific coliphage towards gamma radiation were tested in vitro experiment. The D_10_-values of somatic coliphage and F-specific coliphage in broth medium were 0.59 and 0.58 kGy, respectively, indicating differences in their response to gamma radiation. These D_10_-values were in consistent with the D_10_-value (0.5 kGy) of MS-2 (F-specific coliphage) in distilled or tap water reported by Sommer et al. (2001) and Thompson and Blatchley III (2000). In contrast, Jebri et al. (2013) found higher D_10_-values (1.6, 1.3, and 1.0 kGy) for somatic coliphage, F-specific RNA coliphage, and MS-2 coliphage in raw sewage, respectively. The variation in the response of bacteriophages to gamma radiation could be due to the differences in their structures, size, and nucleic acid (Sommer et al. 2001). From the results of the present work, it is obvious that both coliphages were more resistant to gamma radiation than *E. coli*. This is in accordance with the data of Jebri et al. (2013) who reported that indicator bacteriophages were more resistant to gamma radiation than indicator targeting bacteria. This means that both bacteriophages (somatic and F-specific bacteriophages) required significant greater gamma irradiation doses than their targeting *E. coli*, and somatic bacteriophage required slightest (but significant) greater doses than those needed for F-specific RNA coliphage. Also, our results show that somatic coliphage was a little bit more resistant to gamma irradiation than F-specific coliphage. It was reported that somatic bacteriophage X174 had the most radio-tolerant followed by MS-2 (F-specific bacteriophage) and *E. coli* in sewage and sewage sludge (Sommer et al. 2001 and Gehringer et al. 2003). They added that both groups of bacteriophages required significant higher gamma radiation doses than *E. coli*. In general, it is well known that viruses are more resistant to ionizing radiation than bacteria, and their D_10_-values ranged from 1.0 to 5.0 kGy (Praveen et al. 2013).

The results of the effect of gamma irradiation on the *E. coli* artificially inoculated into minimally processed vegetables (carrot slices, cucumber slices, green pepper quarters, and trimmed watercress), which indicate that gamma irradiation of these products at 2.0 kGy significantly reduced the counts of *E. coli*. The log counts of reduction from the initial counts (8.2–8.9 logs) were 6.0, 5.78, 5.87, and 5.33 for *E. coli*-2 inoculated into carrot spices, cucumber species, green pepper quarters, and trimmed watercress, respectively. Meanwhile, the log counts of *E. coli*-10 reduced by 5.64, 5.33, 5.40, and 4.93 logs, respectively.

The range of log count reduction in both *E. coli* isolates used resulting from exposing these minimally processed vegetables to 2.0 kGy of gamma radiation was between 4.93 and 6.0 logs according to the produce. Other investigators reported that low irradiation dose of 1.0–2.0 kGy greatly reduced the counts of *E. coli* in minimally processed vegetables (Lu et al. 2005; Abu El-Nour Salwa 2007). The mechanism of action of gamma radiation on the killing of microbial cells is due mainly to the direct and indirect effects: direct effect through the photon energy of gamma radiation on the DNA of the microbial cells leading to single or double strand break, while indirect effect is due to the free radicals (H, OH, e) resulting from water radiolysis (Munir and Federighi 2020).

The effect of treatment with individual coliphage, i.e., somatic coliphage and F-specific coliphage on both *E. coli* (2 and 10), indicated that these coliphages greatly reduced the counts of both *E. coli*. The range of reduction from the initial counts (8.2–8.9 log) was between 5.33 and 7.0 logs at the first day of infection. This indicates that the treatment with coliphages was more effective in reducing *E. coli* in comparison with 2.0 kGy gamma irradiation. Similar results have been shown by many researchers. El-Shibiny (2016) found that bacteriophage EC3 reduced the number of *E. coli* in vitro experiment to undetectable limit after 120 min of infection. Application of this coliphage to the surface of cucumber resulted in reducing *E. coli* to undetectable limit after 5 days of storage at 4 °C. Phage cocktail applied on top of tomato and spinach caused 95% and 100% reduction in *E. coli* (Sommer et al. 2001), respectively. Boyacioglu et al. (2013) found that bacteriophage cocktail (Ecoshield™) was effective in reducing *E. coli* 0157:H7 on the surface of fresh-cut leafy green (lettuce and spinach) by 2.49 and 3.28 log units in 30 min and 2 h, respectively. This significant reduction was maintained over the 7 days of storage period at 4 °C.

The reduction of *E. coli* in minimally processed vegetables because of using coliphages could be attributed to the lysis activities of the coliphages that adsorbed onto the surface of the host bacterial cells leading to structural impairment of the cell wall and consequent lysis of the cells (Abedon 2011; Sharma et al. 2017).

During storage of all irradiated or coliphage treated minimally processed vegetable samples under investigation at 4 °C, the survival counts slightly increased to reach almost less than 3 logs_10_ CFU/g even after 9 days of storage. On the other hand, treatments of the minimally processed vegetables samples inoculated with *E. coli* by irradiation preserved fresh produce under investigation to 9 days at 4 °C. The effectiveness of irradiation in preserving fresh produce has been reported by many investigators. Pinela et al. (2016) found that 2.0 kGy dose of gamma radiation preserved the overall quality of fresh-cut watercress during storage period of 7 days at 4 °C. Rezende et al. (2014) reported that irradiation dose of 1.5 kGy kept minimally processed spinach to 12 days at 4 °C and improved its microbial safety. Banerjee et al. (2016) found that irradiation dose of 2.0 kGy extended the shelf-life of ready-to-eat cabbage to 8 at 10 °C, while retaining the microbial and sensory quality.

Combination treatments of coliphages and irradiation maintain the counts of *E. coli* below detectable level over the storage period (9 days at 4 °C). In conclusion, the results obtained in the present study demonstrate the effectiveness of coliphages combined with irradiation to eliminate multi-drug resistance and biofilm-forming *E. coli* inoculated into minimally processed vegetables throughout the storage period of 9 days at 4 °C.

The presence of a biofilm structure could prevent the penetration of phages into a community of bacterial cells. This could be associated with the masking of certain phage receptors owing to the strong binding between individual cells (Rickard et al. 2003). The composition of this structure consists of extracellular polymeric substances (EPS), which serve as an essential base for preserving the stability of the biofilm structure (Felz et al. 2019). According to Bertoglio et al. (2018), the primary constituents of EPS include polysaccharides, proteins, nucleic acids, lipids, and environmental DNA (eDNA).

When implementing this approach in large-scale applications, it is crucial to note that the combined gamma irradiation and coliphage approach was more successful at lowering the concentration of polysaccharides and proteins in the biofilm matrix (Fig. 7). The findings revealed that gamma irradiation and bacteriophages worked together more effectively than they did separately to disrupt the biofilm matrix and increase cell removal. Two steps are thought to be involved in achieving this synergistic mechanism. Gamma radiation can first affect the entire bacterial community by eradicating the bacteria and loosening the biofilm matrix. Gamma radiation causes bacteria to lose their capacity to replicate, which results in cell death. Second, by loosening the biofilm matrix, bacteriophages will be able to pierce deeper into the biofilm and infect the targeted hosts that are embedded closer to the membrane surface. Exopolysaccharides, the primary barrier of the biofilm matrix, have been demonstrated to be degraded by phage-borne enzymes (Knirel et al. 2020). Therefore, phages could eliminate the bacteria in the biofilm when they encounter the biofilm cells. It is also believed that bacteriophages can diffuse through the biofilms’ pores and channels, reaching each of their various layers. Gamma irradiation and bacteriophages can cause the latter to enter lytic mode, as evidenced by the decreased EPS when bacteriophages were introduced independently from gamma radiation; this result agrees with Campbell (2003). Contrarily, Montañez-Izquierdo et al. (2012) claimed that phages frequently do not completely eradicate host bacteria.

According to electrophoretic studies on the bacteriophage virion, the tail fibers are likely positively charged, whereas the head has been hypothesized to be the reason for the phage virion’s negative charge (Perez Esteban et al. 2016). According to earlier research by Morrow et al. (2005), the zeta potential of various *E. coli* strains ranged from − 4.9 to − 29 mV, which is consistent with the observed results in the present study. Accordingly, there may be an electrostatic repulsion that could reduce the effectiveness of contact between both genera, in addition to the interaction between bacteriophages and cell surface receptors. Gram-negative bacteria possess a density of negatively charged outer layers that are basically the result of several forces that require to be stabilized by divalent cations to facilitate bacteriophage adhering to the bacterial membrane. Therefore, Ca^2+^ ions may increase the overall amount of phage particles at the host surface or modify the structure of a cell surface receptor, which causes greater proximity of the receptor molecules and the transfer of phage nucleic acids (Watanabe and Takesue 1972). A possible explanation is that adding CaCl_2_ to the media would increase the distribution of positively charged ions and act as a bridge, which in turn affected the virion surface and the bacteriophage zeta potential. Additionally, it should be noted that these ions, particularly divalent ions, are crucial to the process of bacteriophage infection (Chhibber et al. 2014). This could provide a possible explanation for why the zeta potential decreases when bacteriophage is used, as CaCl_2_ drastically decreases the negativity of bacterial cell surfaces, as shown in Table 2. This confirms that the presence of the divalent ions CaCl_2_, which serve as bridges, reduces electrostatic repulsion between bacterial cell surfaces and coliphages. This is consistent with the Zemb et al. (2013) study, which demonstrated that adding NaCl to freshwater improves the attachment of bacteriophage T2 virions to *Escherichia coli*. Jamal et al. (2015) observed an impressive rise in phage absorption onto host bacteria with the addition of 10 mM of CaCl_2_.

Zeta potential values, on the other hand, were more negative following gamma radiation treatment, which may be related to the radiation’s effects. Sulfur compounds protect the cell wall from radiation, and the lipopolysaccharide’s phosphate groups are responsible for the overall negative charge (Oskouei et al. 2022). It is possible that the effect of gamma irradiation is the reason why combined treatment also reduces cell charge, but not as much as individual treatment with coliphages.

The size values of treated bacterial cells with combined treatment (gamma irradiation + coliphage) are nearly identical to those treated with coliphages (Table 2). On the other hand, the fact that the size of the cells can be determined almost immediately following coliphage treatments and is nearly identical to untreated bacterial cells shows that there was no binding during the measurements. It was supposed that it would take an extended period for the bacteriophages to bind bacterial cells and begin their lytic cycle. It is well known that exposing bacterial cells to ionizing radiation causes additional stress, which tends to disrupt their structure (Young 2006).

Finally, it might be proposed that there are five major categories into which the mechanisms by which phages eradicate bacterial biofilms can be divided: (1) the presence of genes in phages responsible for releasing lytic enzymes that cause the breakdown of the extracellular polysaccharide of biofilms, and the absence of such elements prevents the network connection of biofilms, leading to the destruction of biofilms (Knirel et al. 2020); (2) the lytic enzymes on the phage tail can lyse the bacterial cell wall (Cornelissen et al. 2012); (3) after infecting the host, the phage causes the host to express enzymes that can degrade biofilms (Lewis 2007); (4) the phage can penetrate the biofilms via the hydrophobic channel of the biofilms, destroying the bacteria from the membrane (Briandet et al. 2008); (5) by releasing host quorum sensing inhibitors, the phage impairs communication between bacteria individuals, facilitating the phage’s attacking ability against bacteria (Kaistha and Umrao 2016).

## Conclusion

The present study has demonstrated the efficiencies of coliphages combined with a low dose of gamma irradiation (1.5 kGy) in the elimination of multi-drug-resistant and biofilm-forming *E. coli* inoculated in minimally processed vegetables (carrot, cucumber, green pepper, and watercress). The obtained results also indicated that these treatments could extend the shelf life of the investigated fresh produce to 9 days at 4 °C. Thus, the use of coliphage in combination with gamma irradiation can be applied to improve fresh produce’s microbial safety and enhance its storability in supermarkets.

## Data Availability

All data generated or analyzed during this study are included in this published article.
